# Flexor Carpi Radialis Tendon Rupture in a Distal Radius Malunion

**DOI:** 10.5152/eurasianjmed.2022.21096

**Published:** 2022-02-01

**Authors:** Lauren Seo, Ronit Wollstein

**Affiliations:** Department of Orthopedic Surgery, New York University School of Medicine, New York, USA

## Abstract

We describe a rare case of complete rupture of the flexor carpi radialis in a distal radius malunion. Though the tendon was not repaired, the functional result was acceptable. The coronavirus disease-19 epidemic highlights delayed treatment for routine orthopedic trauma and its complications. Decision-making as to repair or reconstruction should rely on patient characteristics, fracture age, and geometry, as well as the need for other repairs and grafting.

Keywords: Distal radius fracture; flexor carpi radialis; malunion; tendon rupture

## Introduction

Concomitant rupture of the flexor carpi radialis (FCR) with a distal radius fracture (DRF) is rare and has been described in the setting of acute and open fractures.^1^ We present a case of a distal radius malunion left untreated due to the coronavirus 2019 (COVID-19) epidemic with an intraoperative finding of a complete FCR rupture. The late presentation affected the treatment of both the fracture and the FCR tear (the bone since an osteotomy and bone graft were necessary, and the tendon laceration since in an acute setting repair was likely feasible).

## Case Report

A 41-year-old right-handed female presented with pain in her left wrist after sustaining a distal radius fracture 10 weeks prior. She did not receive timely treatment due to fear of exposure to COVID-19. She complained of weakness, pain, dropping objects, and paresthesias in a median nerve distribution.

**Figure 1.**
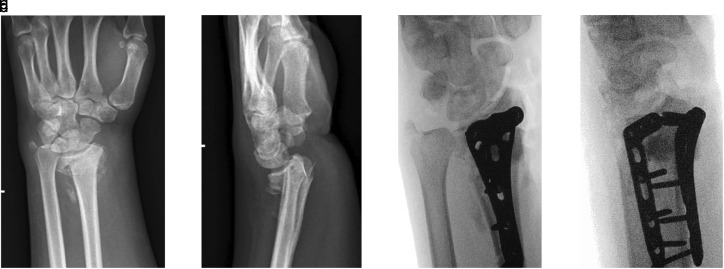
a-d. Pre- and postoperative radiographs of the left wrist with a malunion of the distal radius. (a) Preoperative posteroanterior radiograph of the malunion; (b) preoperative lateral radiograph of the malunion; (c) postoperative posteroanterior radiograph of the malunion; (d) postoperative lateral radiograph of the malunion.

**Figure 2.**
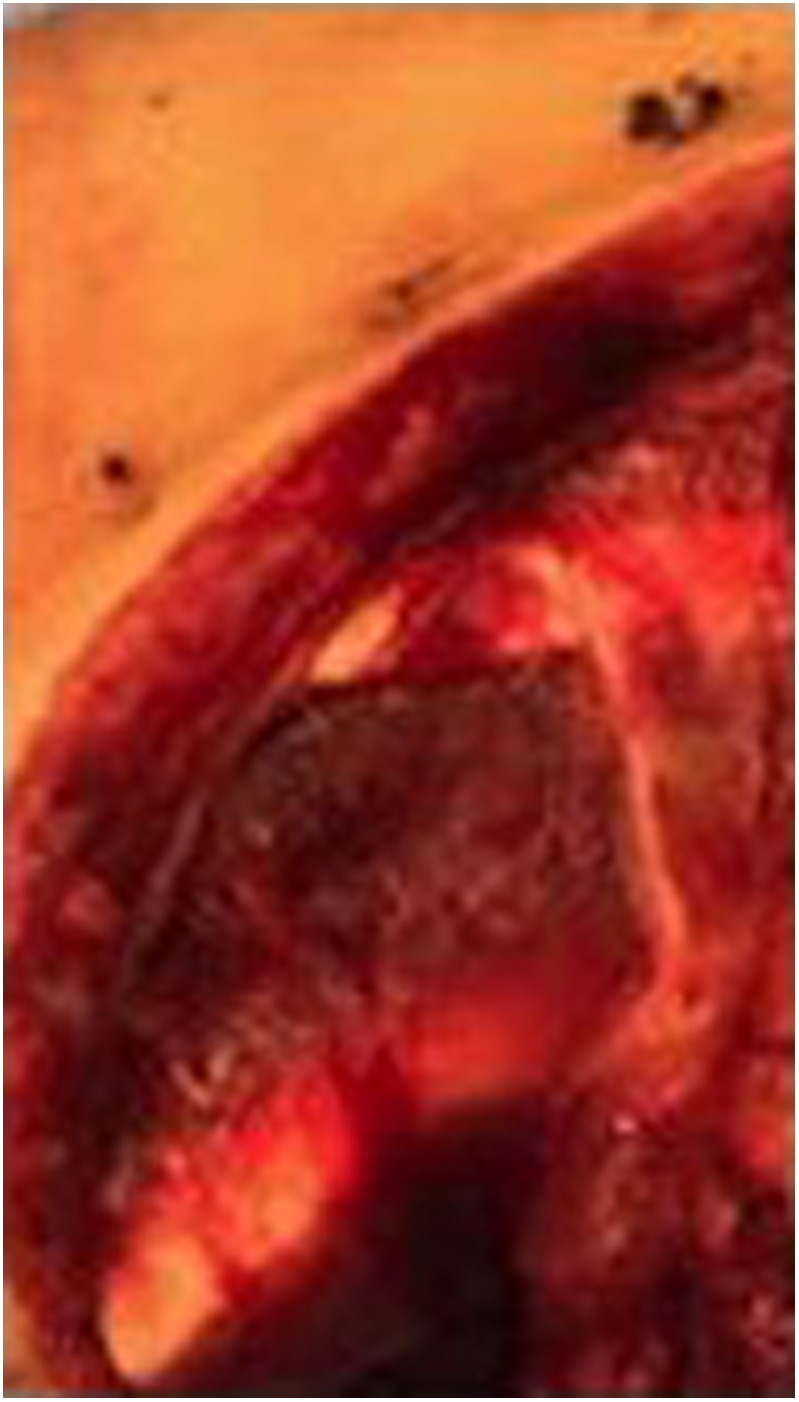
A photograph of proximal flexor carpi radialis tear following debridement.

On examination, beyond swelling and deformity, she had no wrist motion apart from limited wrist flexion. Neurological examination revealed thenar musculature weakness and an 8 mm 2-point discrimination in the median nerve distribution. Radiographs of the left wrist are shown in Figure 1. We unfortunately did not have good-quality photos of the rupture, but the tendon was torn and slightly frayed (Figure 2).

Eleven weeks after injury, an osteotomy and internal fixation of the left distal radius malunion (combined volar and dorsal approach) were performed. The ruptured FCR tendon was identified immediately after the skin was incised. The proximal end of the ruptured tendon was frayed and proximal in the forearm. A partial tear of the flexor pollicis longus (FPL) and extensor pollicis longus (EPL) was also identified as well as injury to the median nerve that was found to be scarred. A carpal tunnel release and neurolysis of the median nerve in the forearm were performed. The FCR tendon was not repaired since (1) loss of FCR function carries minimal morbidity,^2^ (2) reconstruction is complex with minimal gain, and (3) patient was not consented for tendon graft, which should be performed under optimal conditions.

The patient failed to adhere to postoperative rehabilitation, and 2 weeks postoperatively had already removed the cast and was weight bearing on the left hand. Her median nerve symptoms had improved and she had improved motion. Interval radiographs did demonstrate loss of radial height that did not seem to affect the early outcome (Figure 1).

## Discussion

It is possible that the FCR rupture occurred acutely with the tears of the EPL and FPL due to the energy of the fall and the spike in the bone. However, it is also possible that this was an attrition rupture due to the malunion together with the early, unprotected use of the hand and movement of the flexor tendons across the bony spike.

Though the FCR is anatomically distant from the bone, it may have been injured in the acute setting due to the fracture pattern (volar spike) at the time of significant dorsal angulation. The spike in this case was fairly distal, and it is therefore unclear why the tendon was torn so close to the musculotendinous junction. It is possible that this was an anatomic variant with a distal muscle belly that retracted following the tear and was thus identified more proximally at the time of delayed surgery or perhaps the contact with the bone occurred during wrist extension.

A review of the literature reveals 3 reports of acute FCR ruptures with a DRF.^3,4^ Notably, each rupture was an intraoperative finding, and in each case, the tear was ascribed to a prominent volar spike displaced by the fracture. In the report by Erickson et al^4^ the fracture was also associated with a median nerve injury as in this case.^4^ In each instance, the FCR was repaired primarily since it was identified acutely.^4^ Since it is likely technically feasible to repair the FCR in the acute setting, it makes sense to repair the tendon at the time of fracture reduction and fixation.

A report of flexor tendon attrition ruptures following a radius malunion supports reconstruction when there is a small number of tendon ruptures.^5^ Komura et al^5^ concluded that even if not performing an osteotomy for reduction, surgery to shave off the spike would be necessary to prevent additional tendon ruptures. Studies looking at harvesting the FCR have demonstrated no functional loss.^2^ In our patient, given the need for an osteotomy, bone graft, median nerve repair, other tendon repairs, and likely a tendon graft, we decided not to graft the tendon and the patient had a good outcome.

Since at this time we are likely seeing more distal radius malunions as a result of the COVID-19 epidemic, we are likely to see more tendon complications including rare tendon ruptures. In a review of all of the wrist tendon injuries (delayed) seen since March 2020 in our institution, we found 4 cases, none of them associated with a fracture or fracture nonunion. In addition to encouraging patients to apply for treatment in a timely fashion and providing safe healthcare environments, it is important to anticipate tendon injury and the need for grafting in preparing the patient for surgery. The decision of whether to repair or reconstruct should be based on patient characteristics, fracture age, and geometry, as well as the need for other repairs and grafting.
